# Roles and the Moral Practice of Precedent

**DOI:** 10.1093/ojls/gqad020

**Published:** 2023-09-27

**Authors:** Nathan Van Wees

**Keywords:** precedent, stare decisis, legal theory, legal reasoning, moral obligations, roles

## Abstract

Some recent work in legal theory argues that legal questions boil down to moral questions. On this view, lawyers and judges are ultimately interested in the moral effect of things done by legal institutions. This view has been called the ‘new legal anti-positivism’. So far, it has not given a convincing account of precedent. That is, it has not explained how moral reasons can account for what judges do in practice when they follow past decisions. Any successful account must explain the central features of this practice: why lower courts follow higher courts, and not the other way around; the difference between *ratio* and *obiter*; and the situations in which judges distinguish or overrule past decisions. This article gives a non-positivist account that meets this challenge, by giving a prominent place to the moral importance of roles. The account avoids some problems faced by existing non-positivist accounts of precedent.

## 1. Introduction

Some recent work in legal theory argues that legal questions boil down to moral questions. On this view, lawyers and judges are ultimately interested in the moral effect of things done by legal institutions. This view has been called the ‘new legal anti-positivism’.^1^ So far, it has not given a convincing account of precedent. That is, it has not explained how moral reasons can account for what judges do in practice when they follow past decisions. Any successful account must be able to explain the central features of this practice: why lower courts follow higher courts, and not the other way around; the difference between *ratio* and *obiter*; and the situations in which judges distinguish or overrule past decisions.^2^ This article gives a non-positivist account that meets this challenge, mainly by focusing on the moral importance of roles. In doing so, it avoids some problems faced by existing non-positivist accounts of precedent.

My aim, then, is to explain how a decision made by Judge A in an earlier case changes the moral reasons of Judge B in a later case. I start with the scenario in which each judge is a member of the same court. Section 2 explains why one judge has a reason to engage with the past decisions of other judges. Section 3 argues that this requires Judge B to *consider* Judge A’s decision, but Judge B may depart from that earlier decision if it was wrong. Section 4 moves on to the different scenario in which Judge A sits in a higher court than Judge B. Here, Judge B has stronger reasons to follow the decision made by Judge A, whether it was correct or not. Those reasons are based on the moral value which comes from each judge playing her distinct role in the judicial hierarchy.

As I said, I think non-positivist accounts have not yet told a convincing story about precedent. The accounts of Mark Greenberg, Ronald Dworkin, and Scott Hershovitz are all subject to some problems. The latter parts of the article explore these problems and explain how my view avoids them. Section 5 considers Dworkin’s account, and explains why lower courts are bound by higher courts, but the reverse is not true. Section 6 explains how moral thought can distinguish *ratio* from *obiter*, and section 7 briefly concludes. My account, I hope, offers a more compelling way for non-positivists to explain precedent as a moral practice.

## 2. Engaging with the Past: Roles and Agency

How does the fact that Judge A made a decision in a past case give Judge B a moral reason for action in a later case, when each judge sits in the same court? One starting point for an answer to this question is given by Scott Hershovitz, who argues that it is a mark of acting morally that one aims for some kind of coherence in one’s actions and moral attitudes.^3^ As Hershovitz says, we may sometimes be unsure of the correct moral answer to a problem, but we are ‘confident that morality does not demand that we act capriciously or whimsically in matters of importance’.^4^ This can all be understood as a demand that we act with integrity, and that demand gives courts a duty to ‘engage with the past’.^5^

The idea that we should engage with our past has some force for any moral agent, whether a legal official or not. Imagine a doctor called on to perform abortions. He is the only doctor in town, so no other doctor will step in if he refuses. Curiously, this doctor decides afresh each working day whether or not he will assist.^6^ In this scenario, we might accept that the doctor refuses to assist due to his moral views, even if we disagree with those views. But we would not accept that the doctor performs abortions on Mondays, Wednesdays, and Fridays, but conscientiously objects on Tuesdays and Thursdays. And we would find such a stance even stranger if it turned out that the doctor did not even consider what he had done on Monday when deciding what to do on Tuesday.

One reason such behaviour would strike us as odd is that we do not revise our moral beliefs very often. Doing so takes a lot of time. A doctor who thoroughly considers all of the arguments about the moral permissibility of abortion every morning is a doctor who will surely be late to work. The moral considerations at play do not seem to change from day to day, so it seems to make sense for the doctor to take yesterday’s deliberations as a guide for today’s behaviour.

We can express these thoughts by saying that the doctor is ignoring prudential and epistemic reasons to engage with his past. But is the doctor also ignoring *moral* reasons to engage with his past? Perhaps not. If the doctor seriously considers the morally relevant factors each morning, can we really ask any more of him? What morality seems to demand of the doctor, as it demands of all moral agents, is that he seriously consider these moral factors and then act on the view which they support. If that is true, then we have no general moral reason to engage with our past. We can change our mind from day to day, so long as we are careful with our moral deliberations each morning.

In at least some cases, however, we might demand that a person stick to their decision—we might demand, that is, that they do the same thing today as they did yesterday. Moreover, we might think the person is morally required to do this. When can we say a person is morally required to take the past into account in this way? Three considerations seem to be relevant. First, the decision must affects others’ interests in a more or less direct way. Second, a demand of this kind seems more appropriate when we need stability or predictability from the decision maker—because we need to plan our activities around her decisions, for example. Third, the demand will be appropriate when it may be unfair for the person to act differently now than she did in the past. In other words, we are likely to say that a person has a moral reason to engage with their past decisions when there is value for us in stability, predictability, or fairness.

I will return later to the question of what it means to engage with one’s past, and whether it really requires people to act consistently with their past decisions. For now, it is enough to say that moral agents have a reason to engage with their past when stability, predictability, or fairness is on the line, even if they are not necessarily decisive considerations for the decision maker. This is a good starting point for an argument that judges have moral reasons to follow precedent: these three considerations seem to have *some* moral weight for judges when they make decisions, and this makes the past relevant to the present.

Even at this early stage of the argument, however, there is an objection, given by David Lyons.^7^ Lyons says that even if I ought to act consistently with my own past actions, that does not show that I also ought to act consistently with the past actions of others. There is no reason for Judge B to care about Judge A’s past actions. For the actions of Judge A to affect Judge B, we would need a requirement of institutional consistency, rather than merely individual consistency. Lyons asks how we can move from individual consistency to institutional consistency, and his answer is blunt: ‘I see no way of bridging that gap.’^8^

Lyons’ argument is about whether we ought to be consistent across time. So far, I have not argued that we must act consistently. All I have argued is that we ought to consider our own past decisions. But we can reframe Lyons’ point as an objection to my view: the objection is that even if we must consider our own past decisions, we need not consider the past decisions of others.

We can see that this objection has some force by imagining an example in which you and I each have two children. One of your children does some good deed, and you reward them with an ice cream. Your other child now does the same good deed. You now plausibly have a reason to give the second child an ice cream, too. That reason is based on considerations of fairness—moulded, crucially, by your own prior actions. But if one of my children now does the same deed, do I also have a reason of fairness to give them an ice cream, or even to consider the fact that you gave your child an ice cream? It seems not. Facts about your ice cream history do not have any relevance to me and my actions. The moral weight of the past seems to stick close to each individual agent.

This indicates that perhaps Judge B does not have a reason to engage with the past decisions of Judge A, after all. But I think that conclusion would be too quick: there is still reason to think that the actions of Judge A affect the moral reasons of Judge B. This is based on the idea that these two judges are not best seen as two separate moral agents, after all. The better way to think about Judge A and Judge B is this: they are two inhabitants of the same role—namely, *a judge of this court*. Moral agency attaches, in a sense, to that role (or office) rather than to its particular inhabitants.^9^

The basic idea here is a common one: our roles shape our moral reasons in significant ways. Roles are ordinarily nested within broader institutional structures, which set up a division of responsibilities. If we have a reason to carry out the responsibilities of our role in a given institution, that can change the moral relevance of some considerations: some facts may become relevant where they would otherwise be irrelevant, and *vice versa*.^10^

Importantly, this change in the moral relevance of particular considerations will apply to any person who steps into that role. The role itself determines what is morally relevant to us, and what is not. This is why, as Scott Shapiro says, the ‘normative character’ of an office does not change as its occupants change: ‘If X and Y are both eligible to occupy office O, then X and Y will have the same rights and responsibilities when they occupy O.’^11^ Indeed, offices can even exist without occupants: the papacy exists even after a pope dies but before a new one is chosen.^12^ Thus, we can speak about what the new pope ought to do even before we know who he is. The moral reasons he has arise by virtue of his office, not his personal identity.

Another example helps to show how a particular role can take on a moral life of its own. Imagine that Anna has three children, and leaves them in Uncle Toby’s care for a few weeks. Under Anna’s charge, only one child gets ice cream for dessert each week, and then it is the next child’s turn the following week. When Uncle Toby takes over, he declares that there shall be no ice cream in any household under his control.

The children revolt at this idea, and none more so than the unlucky third child, who was next in line. Her indignation can be explained by the fact that Uncle Toby takes on Anna’s role here—the role, let’s say, of *head of the household*. Uncle Toby is not free to make of that role what he pleases. The *head of the household* carries certain moral baggage—namely, at least some of the commitments made by Anna in the past, and some of her moral responsibilities. If a claim of fairness could be made against Anna, it could now be made against Uncle Toby: he will have the same reason to act fairly as Anna previously had. The role is inseparable from the reasons. In other words, the role appears to have a moral form which is (at least somewhat) insensitive to its particular inhabitant.

These ideas also fit many of our ordinary beliefs about judges. We do not think, for example, that the outcome of a case should depend on the identity of the judge hearing it. Rather, we think that *any* judge of a given court will have reason to decide a case in a particular way, given past events. Judges act through their office, and whatever is done by one person in that office affects what ought to be done by someone else acting in the same post. Judges of a court are, in this respect, in the same position as the head of the household: their moral reasons and responsibilities are defined by their role or office, not by their identity. This is what it means to say that the judge has moral reasons by virtue of his or her office. Because the moral world of a judge is shaped entirely by his or her office, moral agency seems to attach to the judicial role itself.

Jeremy Waldron has made similar arguments. Waldron says that each judge makes her decision ‘*as a court*, as part of the judiciary. The order that is issued for the case in front of her is not to be regarded as an order of this particular person; it is an order of the court.’^13^ This is the same court to which the later judge belongs: each is ‘acting in the name of the selfsame entity’.^14^

We often ascribe moral agency to such entities. We speak about a corporation being morally responsible for what it has done, for example.^15^ That corporate entity is composed of individual directors and shareholders. But if the corporation incurs debts, it will still be liable for them even if all of its individual directors and shareholders change. The moral rights and responsibilities of the corporate agent remain the same, despite changes in personnel.

We can apply the same thinking to particular institutional roles. We can say that a given office comes with certain moral responsibilities, shaped partly by what other individuals have done while occupying that office in the past. When someone occupies that office, he or she steps into the shoes of that moral agent and takes on those moral responsibilities.

This solves Lyons’ worry. In my reframing of Lyons’ point, the problem was how the past actions of one agent could be morally relevant to another agent. If judges were different moral agents, this would be a genuine problem: why think that Judge A’s past actions affect what Judge B ought to do now? The answer is that judges of the same court are, morally speaking, the same agent. Each inhabits the same office or role—*a judge of this court*—and that role has its own moral identity. If this is right, then judges have a moral reason to consider what other judges of their court have done in the past. We can now discuss this in more concrete terms: what exactly does it mean for a judge to have a moral reason to ‘engage with the past’?

## 3. *Engaging with the Past: Horizontal* Stare Decisis

There is a common view that *stare decisis* amounts to a requirement to treat like cases alike.^16^ Mark Greenberg adopts just this view, for example:

According to the Moral Impact Theory, considerations of fairness support treating like cases alike, so the fact that a case is resolved in a particular way provides a reason for treating relevantly similar cases in the same way in the future.^17^

Greenberg raises two ideas here: the first is fairness, and the second is the principle that like cases be treated alike. I am going to leave fairness aside for a moment, before returning to it later. I will start, instead, with the bare principle of formal equality: that like cases ought to be treated alike. This principle has been heavily criticised,^18^ and I think that criticism should be taken seriously.

Why is the idea that like cases ought to be treated alike not a sound one? Because, as Grant Lamond says, ‘In individual reasoning we do not normally regard the fact that we decided one way in the past as raising some presumption that we should decide the same way in the future’.^19^ If I donate to one charity collector at the top of the street, I do not then feel obliged to also donate to the charity collector at the bottom of the street.^20^ This is an even bigger challenge than that posed by Lyons. Lyons worried about whether we could move from a requirement of individual consistency to one of institutional consistency. The point here is that there is not even a requirement of individual consistency to begin with. Even if we think of multiple judges of a court as being a single moral agent, no such agent is morally compelled to act as they have in the past. In other words, there is ordinarily no moral demand that like cases be treated alike.

This might strike us as odd. When we are treated differently from the way others have been treated in the past, we often feel that is wrong. One reason for this is that a failure to treat like cases alike can be a failure to respect other values—in other words, there might be some other moral demand lying behind the principle of formal equality. For example, perhaps we ought to treat like cases alike because doing so realises the value of stability and predictability.

Importantly for our purposes, treating like cases alike may also be a way of ensuring that our behaviour is fair. After all, we often describe our treatment as ‘unfair’ when we are treated differently from others. This is a common intuitive response, frequently combined with strong emotions,^21^ and it ought not be downplayed. This idea of fairness might therefore give us another way to argue that like cases ought to be treated alike. That is, of course, how Greenberg frames the argument: it is fairness which supports like cases being treated alike, rather than a free-standing principle of formal equality.

Why do we have a strong intuition that differential treatment is unfair, and what does this tell us? Perhaps nothing at all. Some deny that fairness has any real moral weight: if an original decision was a bad one, for example, there is nothing to be said in favour of repeating it.^22^ This seems, to me, slightly too strong a position. The better view is that fairness does have *some* value.^23^ For example, imagine that A and B are arrested on false pretences and charged with the same crime. A is found guilty while B is not, despite the evidence against them being the same. The major concern here is A’s wrongful conviction, but that is not the whole story: there does seem to be an added wrong (unfairness) in the differential treatment between A and B. Things would have been in one way better if A and B had been treated the same—namely, they would have been fairer. So, there is some value in fairness itself, and this does support like cases being treated alike.

It is clear, however, that this consideration is not very weighty. Although fairness does count in favour of treating B as A was treated, a scenario in which both were falsely imprisoned would clearly have been worse overall.^24^ The value of fairness does not justify repeating the moral error. In other words, fairness will probably be insufficient to justify treating like cases alike.

Fairness may be more weighty in some cases than others, of course.^25^ In the example above, the question is whether fairness justifies harming B given that A has been harmed—and harmed, we may add, in a very significant way. But other cases will involve benefits, rather than harms. Imagine that X is sentenced to five years’ imprisonment, when he should have been sentenced to six years. Does fairness demand that we now give the same benefit (ie lenient sentence) to Y, who committed the same crime in the same circumstances? The reason of fairness which supports like treatment seems stronger here than in the previous example, because there is no risk of causing additional unjust suffering. But there may also be good reasons to sentence offenders of this type to six years’ imprisonment, and we would need to know more before concluding that fairness could overcome those (presumably weighty) reasons. So, fairness might counsel giving the same sentence to Y as was given to X, but it is hard to say that it compels it.

If the value of fairness is often so small, why does the perceived unfairness of differential treatment often provoke significant anger? Why might A feel so aggrieved at B being rightly acquitted in our original example? Joel Feinberg suggests that our anger stems from the fact that the principle that like cases ought to be treated alike ‘is a principle of reason, in much the same way as Aristotle’s principles of identity, contradiction, and excluded middle are “laws of thought”’.^26^ So, treating similar cases differently ‘offends against impersonal reason itself’.^27^ Our anger at being treated differently (unfairly) is therefore increased because it ‘borrows some of the authority of reason’.^28^

This indicates that where there is unfairness, there is at least suspicion of treatment without good reason. (This is why we are often on the lookout for discrimination based on things such as race or sex when we see unequal treatment.) What is needed to combat this suspicion of treatment without good reason? Good reasons, of course: we need to explain to a person why, despite unfairness in his differential treatment, there is still good reason for that differential treatment. And this makes sense, I think, of an idea put forward by David Strauss. Strauss says that we can understand our demand for fairness precisely as a demand for reasons: it is a ‘demand that differences in treatment be justified by [good] reasons’.^29^ Those reasons will be good ones, it seems, if they can overcome whatever unfairness is involved in departing from our past actions.

We can see, then, why judges have a reason to engage with their past, and what this means. It does not mean a requirement to treat like cases alike. Rather, it means considering what has been done in the past, and offering good reasons for what ought to happen now. Those considerations which favour sticking with the same course—fairness, predictability, and stability—have real moral value, and therefore must be considered. But these may be outweighed by other considerations which favour a change of course. In explaining why there are good reasons for change, a judge meaningfully engages with the past and, in doing so, takes seriously our demand for fair treatment.

I now want to put this into more concrete terms: what should Judge B do in the face of Judge A’s earlier decision? This depends on whether Judge A got things right the first time around, so Judge B might face one of three scenarios. First, she may face a decision of Judge A which was correctly decided. Second, she may face a precedent wrongly decided. Third, she may face a precedent which was not completely determined by reasons—one in which there was no uniquely correct decision to be reached. I will discuss these three scenarios in turn.

Where Judge A’s earlier decision was correct, Judge B now ought to make the same decision for the same underlying moral reasons. Those reasons justified the earlier decision and, all else being equal, will justify the same decision again. There are also additional reasons for making the same decision in the later case given by considerations of stability, predictability, and fairness. We have seen already that fairness adds little. Stability and predictability generally have value because they allow people to make plans.^30^ (Their value fluctuates, of course: stability of a bad state of affairs has no value—subject to one caveat discussed below—and may be more valuable in some contexts than others.^31^) While these considerations count in favour of repeating the earlier, correct decision, they can be seen as superfluous: the decision would still be correct even if those further considerations were absent.

The second scenario supposes that Judge A’s earlier decision was wrong.^32^ Here, Judge B should not follow Judge A’s earlier decision, subject to some exceptions discussed below. This is because, as we have seen, engaging with your history does not require you to repeat the errors of your past. What we can demand of moral agents is that they take their past into account, and offer up good substantive reasons for what they intend to do now. Here, Judge B has good reason to depart from what Judge A did earlier: namely, that the morally relevant factors support acting differently.

This still leaves space for the idea that Judge B has *some* reasons to follow Judge A’s decision. These are based on considerations of fairness, stability, and predictability. I will discuss the latter two in a moment. As for fairness, we have already seen that its value, though real, is minimal. It gives some support to repeating one’s errors, but not enough to justify that course all things considered: we ought not wrongly imprison B just because we wrongly imprisoned A.

It is important to emphasise that Judge B must still consider Judge A’s earlier decision. She is not free to simply ignore it. She must consider whether there is merit in Judge A’s approach, and she ought not decide too hastily that Judge A was wrong. There are costs in stability, predictability, and fairness in departing from the earlier decision, so there is some epistemic burden to be met before Judge B forges a new path. Judge B must explain her conclusion and the reasons which favour taking a different path now. As we have seen, that is necessary in order to take seriously our demand for fairness. And, as Hershovitz points out, this matches what we see in real life: judges do not treat *stare decisis* as a blanket injunction to do what was done in the past. Rather, they treat it as requiring them to follow *or* distinguish *or* overrule previous cases.^33^ In this way, they meet the demand on moral actors to engage with their past, even if they depart from it.

There may be limits to judges’ ability to depart from previous decisions, of course. As we saw in the case of the doctor, we can sometimes legitimately demand that someone stick to a fixed course of action. This will be appropriate where the decision affects other people who need some stability or predictability in the matter. When judges repeatedly change their mind on the same question—judicial ‘flip-flopping’—the loss of stability and predictability may come to have more moral weight than whether the right answer *ex ante* is *a* or *b*.

Judicial flip-flopping is, thankfully, likely to be relatively rare. Judges are all products of the same legal system and legal education. As a result, they tend to share the same outlook on the law, making agreement likely.^34^ Legal systems also have ways of minimising disagreement, and that is by appeal to a higher court—a matter discussed further in section 4.

Nevertheless, flip-flopping is always a possibility. When it does happen, stability and predictability may favour sticking to a given answer even if it is the wrong one *ex ante*. This, of course, is a fuzzy statement: *when* will instability and unpredictability justify sticking with a bad decision? I do not believe this question can be answered in the abstract.^35^ As with all moral matters, context is king. My point is that it is *possible* for these factors to be determinative, and that this mirrors how we ordinarily think about moral decision making outside the legal context. So, if Judge A’s decision was mistaken, Judge B ought to follow it only when stability and predictability come to have conclusive weight. Otherwise, morality demands only that she give good reasons for departing from the earlier, mistaken decision.

What about the third possible scenario, in which Judge A’s decision cannot be said to be either right *or* wrong? This might come about because some questions do not have uniquely correct moral answers, or because we cannot be sure of those answers even if they exist. Law occasionally raises questions of the first kind, and Andrei Marmor gives some examples of this. First, some cases may be based partly on arbitrary choice: there may be reasons to sentence a criminal to a period of imprisonment of between nine and 11 years, but no reason to select any one sentence within that range as opposed to any other.^36^ Second, some cases may involve incommensurable values: there may be no answer as to whether economic efficiency is more valuable than environmental protection in resolving a particular dispute, for example.^37^ Third, some cases may involve ‘moral vagueness’: a case may turn on whether behaviour was ‘dishonest’, but honesty may be a fuzzy concept at the edges.^38^

Some may think the second and third of these categories are based on philosophical errors—values may be commensurable, and moral concepts may have more-or-less hard edges. I am not going to dive into such large questions here though, because Marmor’s first category certainly seems plausible: legal questions do sometimes invite choices which seem arbitrary to some extent. Criminal sentencing is a good example of a case in which reasons can take one only so far. A number must be produced, but the reasoning process cannot be mathematical.^39^

What happens when Judge A’s decision falls into this category? Imagine that Charles was sentenced to *x* years’ imprisonment for committing a crime in particular circumstances. Camilla now commits the same crime, in exactly the same circumstances. If Camilla is sentenced to *x*+1 years, she will feel this is unfair. If she demands good reasons for her differential treatment, I do not see how they could be given. By hypothesis, there are no considerations which distinguish Camilla’s case from Charles’, and the original decision did not weigh the relevant considerations incorrectly. Fairness ordinarily has only minor weight, but in a situation such as this, that weight seems decisive: if there is nothing to be said in favour of one sentence over the other, fairness breaks the tie in favour of sticking with the sentence given to Charles earlier. Engaging with one’s history therefore can require judges to act on the basis of fairness in exceptional cases like this, and therefore to treat like cases alike.

Note the word *exceptional* here. While sentencing decisions happen all the time, it is rare for two cases to be identical, with no morally relevant differences. Nor, I suspect, are there many legal questions quite like criminal sentencing, where reasons seem not to fully determine the answer. (Calculating civil damages for unquantifiable loss is the most obvious further example here.) Sentencing requires morally relevant factors to be converted into a numerical value, and that is an obscure task. It is unlike the types of questions which usually exercise the minds of lawyers and law students—Should A win, or should B win? What constitutes a valid contract?—which are determined by reasons in a more straightforward way, even when they are difficult.

The account I have given here requires judges to engage with their past decisions, but this does not often constrain them to follow those past decisions. Past decisions should be followed when they are correct, for the same reasons that made that earlier decision correct. When they are incorrect, they should be followed only where stability and predictability come to be more important than getting things right. Past decisions should also be followed where earlier decisions were neither right nor wrong—but, as I have explained, such occasions may be relatively rare.

This is what it means, then, for a judge to have a moral reason to engage with the past in these various scenarios. Are these constraints too weak? They certainly do not bind judges to the past in the strict way that some models of *stare decisis* claim that judges are bound. It is not clear to me, however, what values could bind judges in a stronger way. As I have explained, there is no principle of formal equality or fairness which convincingly counsels the repetition of mistakes. Stability and predictability may occasionally count in favour of persisting in a mistaken course, but these are also usually weak considerations. They will carry the day only on the rare occasions of judicial flip-flopping.

I think this theory fits the facts of judicial practice in some major common law jurisdictions. Judges in those jurisdictions do not consider themselves strongly bound by decisions made by other judges of the same court. In Australia, it is said that ‘The doctrine of *stare decisis* does not … compel the conclusion that a judge must always follow a decision of another judge of the same court’.^40^ The later judge may depart from an earlier decision she considers wrong.^41^ In Canada, ‘a departure [from a previous decision] is authorized where a judge is convinced that the prior decision is wrong and can advance cogent reasons in support of this view’.^42^ The conclusion that the earlier decision was wrong is not to be reached lightly.^43^ Importantly, courts recognise that ‘the [later] judge does not start writing on a blank page’.^44^ The judge must consider what has gone before, decide whether it was correct, and then decide whether there are good reasons to now act differently. This matches the theory I have set out.

Things are, admittedly, more difficult for my view in the United Kingdom (UK). The UK Supreme Court may depart from a previous decision where it appears right to do so,^45^ though it is not ordinarily enough that the Court considers the earlier decision to be wrong.^46^ The Court of Appeal similarly considers itself bound by its previous decisions.^47^ That approach is clearly more restrictive than in Canada and Australia, and has indeed been rejected as too strict in New Zealand.^48^ In the latter country, a court will ordinarily follow its earlier decisions but remains free to ‘review and affirm, modify or overrule an earlier decision where it is satisfied it should do so’, with no hard-and-fast rules about when that test is met.^49^ In light of all that, I think my theory holds up well enough against much of common law practice.

## 4. *Vertical* Stare Decisis

For those who are still concerned that my theory allows judges too much freedom, recall that I have so far only discussed the scenario in which Judge A and Judge B are members of the same court. Judges do often feel themselves to be more strongly bound by past decisions when these come from higher courts, and this ‘vertical’ form of *stare decisis* is what I will discuss now.

My explanation of horizontal *stare decisis* focused on the fact that Judge A and Judge B shared the same role, or office. Now that we have elevated Judge A to a higher court, we need a different explanation: the role of *supreme court judge* is not the same as the role of *local court judge*, so we cannot plausibly think that each judge shares in the moral persona of the same office. Rather, the offices of each judge have been deliberately separated in a hierarchical way. In this situation, the best explanation of why Judge B has reason to follow the decisions made by Judge A is based on considerations of the division of labour between their different institutional roles.

It is no accident that the judiciary is separated into different roles.^50^ Much of the work of governing is best done by breaking that work down into different tasks, allocated to different institutional roles. One reason we separate roles in this way, I believe, is that it allows government as a whole to respond to different moral considerations, or types of value. Consider the fact that we have reasons to prosecute people, and reasons for them to be judged impartially before being punished. We would be unlikely to respond well to these two sets of reasons if we allowed one person to do both tasks. The reasons for prosecution would likely dominate that person’s mind, and she would fail to respond to the moral reasons for impartiality in judging that person. It is simply too difficult for one mind to do these two jobs well. Doing a job ‘well’ here, I emphasise, means doing that job in a way which responds to the reasons we have for doing it in the first place—the reasons we have for establishing the relevant role.

This idea underlies the separation of judges into different courts and the placement of those courts into a hierarchy. This structure allows the court system, as a whole, to respond to diverse moral values, which would be impossible if judicial roles were not so divided. These distinct values fall into two categories, and they will not surprise the reader. On the one hand are considerations of fairness, predictability, and stability, and on the other hand are substantive considerations about how certain cases are best resolved (‘accuracy’, for short). Any discussion of precedent must explain how a judicial system can respond to both sets of moral demands: stability and accuracy each have genuine moral force, and neither can simply be ignored.

A hierarchical judicial system is designed to respond to these competing values. Imagine for a moment that the judicial system was not arranged hierarchically, and that each judge sat within a single court. A judge in such a system ought generally to decide cases in accordance with their merits, as I explained in section 3. This leads, as I also explained there, to a risk of judicial flip-flopping: repeated inconsistent decisions made by different judges. This is a realistic possibility, given that horizontal constraints of precedent are relatively weak.

As I said in section 3, flip-flopping may make it necessary to stick to one decision, so as not to lose the value of stability and predictability. But whose decision should we take as the decisive final word? We could wait for judges to agree amongst themselves as to whose word shall be final on a given question, but there is no guarantee that they would reach that agreement any time soon (or at all). A better way to solve the problem is to divide judicial labour between different roles, arranged hierarchically. We can give the courts further up the hierarchy the task of resolving disagreement about the substantive merits of legal disputes. In order to facilitate this task, those superior courts are staffed with our best judges. These judges will generally sit in multi-member benches and, in that way, their talents will be combined. This should lead to better decisions about the substantive merits of each case—in other words, we expect that staffing the superior courts in this way will allow those courts to carry out their allocated task well.

Having given the highest court the task of making decisions which are substantively right, the judicial system can then respond to the moral demand for stability and predictability by giving lower court judges the task of following those decisions. The hierarchical system as a whole therefore responds to diverse moral demands (accuracy and stability) which may pull in opposite directions. So, judges in lower courts have moral reasons to follow decisions made by those above them.

Note that lower courts do not have this reason because the superior court necessarily got things substantively right. As a matter of fact, the higher court may have got things wrong—it, too, is fallible. But lower courts will still have a reason to follow those wrong decisions. This is because doing so is the only way to maintain the judicial hierarchy. And, as we have seen, there are good moral reasons to have that hierarchy in the first place. So, lower court judges have good reasons to carry out their role in that hierarchy, and this does not depend on whether the higher court’s decision was right or wrong.

Interestingly, the points I have made here reverse a common argument for *stare decisis*. It is sometimes said that higher courts have greater expertise, and that lower courts therefore have a reason to follow their decisions. But we should be able to see now that this puts things the wrong way around. We do not start with a talented court and then suppose that others ought to follow it because its decisions are probably right. Rather, we start with the idea that it is good to have a court which others ought to follow, because there is moral value in such a hierarchical system. And if others ought to follow this court, then we should make it the best court we can—a court as likely as possible to make good decisions. To achieve that, we give this higher court the best judges we can find, and make them work together in larger benches.

So, putting better judges at the top of the pyramid does not *produce* reasons for lower court judges to follow their decisions. Rather, it *responds* to the fact that we have reasons to establish a hierarchical system in which lower courts follow decisions from higher courts. It responds to that moral fact by trying to ensure that the decisions made at the top are, in fact, good decisions.

My argument here has been that there are good reasons for courts to be arranged hierarchically, because doing so allows the judicial system to respond to diverse moral values. Those values are realised only so long as lower court judges respect their role, and the role of those above them. In concrete terms, that means that lower court judges ought to follow their superiors’ decisions. We can see, then, that Judge B will now be much more tightly constrained to follow the earlier decisions of Judge A. Only by following those decisions can Judge B play her part in the hierarchical judicial system, and only by adopting and maintaining such a system can the courts (as a whole) respond to the diverse moral values to which we want them to respond. So, Judge B should follow the decisions of Judge A in virtually all situations, leaving an exception only for truly evil precedents.

Before moving on, an objection is worth considering.^51^ I have said that a judicial hierarchy allows the courts to better respond to the demands of stability and predictability by strictly binding lower courts. But does this place too much weight on stability and predictability, forcing lower courts to endlessly repeat bad decisions? Do stability and predictability really have that much moral weight?

I do not think my theory places excessive value on stability and predictability. As I have said, the system I envisage is set up to ensure that the decisions which are followed in lower courts are, in fact, likely to be substantively correct. There is no sacrifice of accuracy to stability and predictability here, so much as an attempt to reconcile these two sets of values.

We also need to remember the kinds of cases which apex courts typically decide, thereby binding lower courts. These are cases where stability and predictability are usually very valuable, such that we want them to be closely followed at lower levels. These cases tend to fall into three categories. The first category covers cases which concern questions of public importance, such as constitutional questions. These typically affect the structural relationship between branches of government, or their powers.^52^ Stability is at a premium, I would suggest, when such questions are on the line. The state cannot carry out its functions, for example, if there are inconsistent decisions on whether a given body has power or responsibility over a particular matter. Decisions on such fundamental questions warrant strong entrenchment in lower courts, even if they are wrong *ex ante*.

The second category of cases are those which have generated persistent disagreement in lower courts.^53^ The third category of cases are those which concern novel and more complex legal questions. This novelty and complexity makes these cases likely to generate disagreement, meaning that they will probably shift into the second category over time. When there is such disagreement, or a real risk of it, we need decisive resolution—in other words, we need a settlement in the name of stability and predictability. As I have explained, this is best achieved by decisions made in higher courts being strictly followed in lower courts.

Apex courts are therefore more likely to deal with cases where stability and predictability are especially important.^54^ This means that when they make decisions on these questions, there are especially strong reasons for courts below to be bound by them. To this extent, at least, a theory like mine—with strong vertical *stare decisis*—does not overvalue stability and predictability. Instead, it gives them their proper weight.

## 5. *Dworkin and the State*

The theory I have set out is similar, in some respects, to what others have said about precedent under the banner of new legal anti-positivism. Here, I want to compare my account to that given by Dworkin and later adopted by Hershovitz. This comparison shows, I think, the value of focusing on roles in the way I have.

The Dworkinian explanation of precedent begins with the idea of the state or community as a whole—‘state’ and ‘community’ being used interchangeably by Dworkin in the relevant passage.^55^ Dworkin’s state or community is ‘some special kind of entity distinct from the actual people who are its citizens’, and he ‘attributes moral agency and responsibility to this entity’.^56^ Thus, the community itself ‘can adopt and express and be faithful or unfaithful to principles of its own, distinct from those of any of its officials or citizens as individuals’.^57^

Dworkin thinks that we engage in this kind of personification of the community when we argue about the political rights we have against the state. We start from the idea that individuals have, for example, a right to be protected against assault. The state bears the corresponding duty to protect us from assault. Importantly, we ‘can debate the scope of the community’s duty [to protect us] and leave for separate consideration the different issue of which arrangement of official duties would best acquit the communal responsibility’.^58^ On this approach, ‘our conclusions about the group … [are] in every way prior to any conclusions about individuals’.^59^ We cannot explain ‘the special responsibilities of political office’ without the idea

that the community as a whole has obligations of impartiality [for example] toward its members, and that officials act as agents for the community in acquitting that responsibility. Here … we need to treat group responsibility as logically prior to the responsibilities of officials one by one.^60^

For Dworkin, integrity is the central moral requirement when thinking about the law, and it is one which falls on the state itself. If we want to know what individual officials ought to do, we have to start with that state-level obligation.

If we accepted this idea, it would indeed explain why the actions of one judge affect what another judge ought to do. We already know that what a moral agent has done in the past affects how that same agent must act now. On Dworkin’s view, each judge is a representative of one such agent—namely, the state. The actions of each judge constitute part of the history of that single moral agent. As Hershovitz explains, this leads to a requirement of *stare decisis*:

Courts are moral actors, and a court can display integrity in much the same way that an individual can. A court displays integrity when its decisions reflect a commitment to a coherent and defensible view of the rights and duties people have under the law. Such a commitment can only be displayed by a pattern of decisions across time.^61^

Hershovitz talks about courts as the relevant moral actors here, but makes it clear elsewhere that it is the state which ultimately matters: it is the state which we want to act with integrity, he says, ‘and, *derivatively*, its courts’.^62^

This is similar to my own view in some ways. There are significant differences, however, in the location of moral agency and the place of roles within the explanation. For Dworkin and Hershovitz, moral commitments are shared by the state as a whole. We figure out the state’s obligations *before* we figure out what is required by people in particular roles. Roles come last on this Dworkinian picture. By contrast, they come first on mine. What Judge B should do depends on her role, and the role of Judge A: do these judges share an office, or are they playing different roles? In my picture, moral agency attaches to particular roles, rather than the state as a whole.

Is my focus on roles more promising than the Dworkinian picture? I think so, primarily because focusing on roles better fits our ordinary thinking about officials’ responsibilities. Institutional roles are more often the starting point for such thinking, rather than the end point. We are used to thinking about what judges ought to do, and we recognise that this is very different from what a legislator ought to do, for example, even though each is a part of the state. So, we divide our thinking based on institutions and the roles they contain. My account matches this way of thinking.

It seems difficult, by contrast, to begin with the state. An example given by Hershovitz himself in another piece seems to show this.^63^ Hershovitz imagines a county clerk who is asked to issue a marriage licence to a same-sex couple. The clerk refuses, based on a provision in the state constitution. A District Court judge orders that the licence be issued, however, because of a federal constitutional provision. Hershovitz uses this example to show that these individuals may all be acting on a fair view of ‘what the law requires’, even though they act to opposite ends. This is possible, he says, because what the law requires may indeed differ depending on one’s perspective.^64^ One’s perspective, we can add, will be defined by one’s role.

If we strip away talk of ‘the law’ here, we are left with this idea: what one has reason to do depends on one’s role. If that is so, it seems odd to start from a view about the obligations of the state as a whole. What is the obligation of the state in the marriage licence example? I suspect that Dworkin would say that the state’s obligation is to issue the marriage licence. But the point of the example is that the county clerk has precisely the opposite reason: she has conclusive reason to deny the licence. Why? Because she acts in a *role* in which it is not appropriate for her to resolve conflicts between the state and federal constitutions. The example therefore shows the virtue of my approach over the Dworkinian approach: we can track the moral reasons of the clerk and the judge more accurately and easily by thinking about roles, rather than by thinking about the state.

Consider another example which shows the benefit of focusing on roles. If there are many lower-court decisions on a given point, then considerations of stability and predictability favour sticking to those decisions in future. These moral considerations seem to apply to all judges. That might lead us to think, then, that higher courts could be bound by the moral weight of those lower court decisions. That would be a bad account of our legal practices, of course: lawyers know that *stare decisis* does not work ‘upwards’ in this way.^65^

How can we rule out faulty accounts of *stare decisis* such as this one? The easiest explanation focuses on roles. As I said in section 4, we have good moral reasons to set up a hierarchical judicial system. The role of judges at the top of that system is to get things substantively right. To play that role well, those judges ought not consider themselves bound to follow decisions made below them. Doing so would get in the way of reaching the substantively right outcome, and so prevent the higher-court judge from fulfilling her role in the judicial hierarchy. We would then miss out on the value which comes from having a hierarchical court structure. So, there are good moral reasons for higher courts *not* to be bound by lower court decisions. Those reasons are based on the distinct roles played by each judge in the court structure.

In this way, roles seem crucial to the best explanations of precedent, so Dworkin has to accommodate them somehow. He does seem to agree, of course, that the precise obligations of the state should be formulated and understood in terms of roles in the end. What is nevertheless important, Dworkin may reiterate, is that we start with the idea of the state, even if we end with the idea of roles. And why is it so important to start with the state? Perhaps because we have a strong intuition that government *as a whole* must treat people fairly.^66^ We can start with this idea, and then figure out exactly what this looks like for particular judges dealing with precedents.

This Dworkinian route is more complex than my own. It requires us to move from moral arguments about the state to moral arguments about particular officials. The marriage licence example showed that this is not always easy. The easier route is, as I have said, to focus on roles from the start. The only benefit claimed for Dworkin’s added complexity is that it gives a better account of the role of fairness in government. This seems doubtful to me: my view has also explained why, and to what extent, judges ought to treat people fairly, and what this looks like in concrete terms.

Dworkin’s approach will reach the right outcome in many cases, and so it is not to be dismissed lightly. Nevertheless, it takes a longer and less certain route to that outcome. Focusing on roles gives a more straightforward explanation, tells us what fairness looks like in different circumstances, and easily accounts for the difference between horizontal and vertical *stare decisis*.

## 6. Ratio *and* Obiter

The difference between *ratio* and *obiter* is a key distinction in the common law practice of precedent. Sebastian Lewis has said that accounts of precedent like mine, which focus on the moral reasons which underpin *stare decisis*, cannot account for that distinction.^67^ I think moral thought can make that distinction. Showing this requires us to think more about what it means to ‘follow’ a decision.

When we say that lower courts have reasons to ‘follow’ decisions made above them, that language can only be a shorthand. As should be clear from section 4, the moral demand on lower courts is to make decisions which meet the need for stability and predictability. That is what we mean, in complete terms, when we say that lower courts must follow decisions made by higher courts.

The question, then, is how lower courts can acquit their moral responsibility to make decisions which meet this need for stability and predictability. In ordinary, non-legal contexts, we would express a desire for this by asking someone to ‘be consistent’. A demand for consistency is usually a demand that we not change our conclusion about a fixed set of moral considerations: if considerations *a*, *b*, and *c* led to outcome *x* yesterday, then we should decide that *a*, *b*, and *c* lead to *x* again today. If we want to demand that someone now act consistently with their previous decision, then, we need to be able to describe that original decision in a way that picks out the considerations which were morally relevant to it. That is, the description needs to pick out *a*, *b* and *c* as relevant, but exclude consideration *d* if it was irrelevant.

I think the same idea holds in the legal context. The distinction between *ratio* and *obiter* is best seen as the distinction between the considerations which were morally relevant to resolution of the earlier case and those which were irrelevant. The *ratio*/*obiter* distinction thereby helps us to know what we have to do in order to act ‘consistently’ with that earlier decision.

Of course, there is often debate about what is part of the *ratio* of a case and what is not. This reflects the fact that it is often difficult to describe an action—including a judicial decision*—*in a way which captures its morally relevant aspects. John Mackie gives a good example of this phenomenon:

Even if *X* is in itself wrong or bad or illegal or dishonourable, it does not follow that *X*-rather-than-*Y* is so. It may be foolish, or a breach of contract, or professional misconduct for a captain to throw cargo overboard in ordinary circumstances, but it may be a wise, justifiable, and commendable action when the ship would otherwise sink.^68^

Similar problems arise with Kant’s ‘formula of universal law’. That formula may deem the same act permissible when described under one maxim but impermissible when described under another.^69^ Everything depends on how well the maxim captures the morally relevant considerations: is it best described as *X*, or is it *X*-rather-than-*Y*?

So, we must often be careful in moral thought to describe actions in ways that fully capture their morally relevant aspects, while excluding their morally irrelevant aspects. My point is that any debate about what is the *ratio* in a case and what is *obiter* is simply a specific example of this more general issue in moral philosophy: it is disagreement about whether some consideration is morally relevant or not, and therefore whether it is properly included in a description of the earlier court’s action. The distinction between *ratio* and *obiter* is therefore an example of a common philosophical problem—difficult to solve in some cases, but by no means novel or insurmountable.

Finally, note that the text of Judge A’s decision is not canonical as to what counts as morally relevant and what does not. As we know, later judges pay attention to what was done in a previous case, rather than what was said.^70^ This reflects the fact that we need to find the best moral description of what was done by Judge A. Importantly, Judge B may be just as capable of giving that description as Judge A, and that is why the text of Judge A’s decision is not canonical in this respect.

## 7. Conclusion

I have explained how the actions of Judge A affect the reasons which Judge B has in a later case. This will depend, I argued, on the particular role of each judge. Where each is a member of the same court, Judge B has a reason to consider the past actions of Judge A. Where Judge B is instead in a lower court, she will have reason to follow Judge A’s decision. In common law terms, this translates into a relatively weak version of horizontal *stare decisis* and a stronger vertical version. This matches the facts: judges do feel more strongly bound by decisions made above them than by decisions made alongside them. Most importantly, my explanation of these facts avoids some of the problems faced by other non-positivist attempts to explain precedent.

One final point is in order. Some may think my account flawed, because the practice of *stare decisis* must require judges to do what is wrong. The practice would be empty, so the thought goes, if it simply instructed judges to do the right thing.^71^ Telling someone to ‘do the right thing’ does not constrain them in any way, and the nature of *stare decisis* is that it constrains.

But trying to justify a practice which constrains judges to act wrongly is a project doomed to failure: by definition, there can be no justification for acting wrongly. So, the effect of judicial decisions cannot be to constrain judges to do the wrong thing. Rather, the effect of judicial decisions is to change what *counts* as being the wrong thing for a judge to do—and, by the same token, what counts as the right thing for that judge to do. I have tried to explain here how that change can come about.

## Footnotes

1 So named by Hasan Dindjer, ‘The New Legal Anti-Positivism’ (2020) 26 Legal Theory 181. The most influential contributions to this way of thinking have been given by Ronald Dworkin, *Justice for Hedgehogs* (Harvard UP 2011) ch 19; Nicos Stavropoulos, ‘Obligations, Interpretivism and the Legal Point of View’ in Andrei Marmor (ed), *The Routledge Companion to Philosophy of Law* (Routledge 2012); Mark Greenberg, ‘The Moral Impact Theory of Law’ (2014) 123 Yale LJ 1288; Scott Hershovitz, ‘The End of Jurisprudence’ (2015) 124 Yale LJ 1160.

2 On this ‘minimum descriptive requirement’, see Sebastian Lewis, ‘On the Nature of Stare Decisis’ in Timothy Endicott, Hafsteinn Dan Kristjánsson and Sebastian Lewis (eds), *Philosophical Foundations of Precedent* (OUP 2023) 36 fn 6; Katharina Stevens, ‘Reasoning by Precedent—Between Rules and Analogies’ (2018) 24 Legal Theory 216, 218–19.

3 Scott Hershovitz, ‘Integrity and Stare Decisis’ in Scott Hershovitz (ed), *Exploring Law’s Empire* (OUP 2006) 114.

4 ibid.

5 ibid 113, 118.

6 The doctor may instead make a plan in advance as to whether he will assist. This plan may be morally problematic if it involves assisting on some days while conscientiously objecting on others. But this example would not tell us much about precedent. I am interested in how one should decide now, given past decisions—not in how one should plan for the future. Some think that precedent also raises questions about future decisions, but, for reasons not relevant here, I am not so sure. See Nina Varsava, ‘The Gravitational Force of Future Decisions’ in Endicott, Kristjánsson and Lewis (n 2).

7 David Lyons, ‘Formal Justice, Moral Commitment, and Judicial Precedent’ (1984) 81 Journal of Philosophy 580, 585–6.

8 ibid 586.

9 I will use the terms ‘role’ and ‘office’ interchangeably here.

10 On these points, see TM Scanlon, *What We Owe to Each Other* (Belknap Press 1998) 52; TM Scanlon, ‘Reasons: A Puzzling Duality?’ in R Jay Wallace and others (eds), *Reason and Value: Themes from the Moral Philosophy of Joseph Raz* (OUP 2004) 241.

11 Scott Shapiro, *Legality* (Belknap Press 2011) 209.

12 ibid 424 fn 12.

13 Jeremy Waldron, ‘Stare Decisis and the Rule of Law: A Layered Approach’ (2012) 111 Mich L Rev 1, 16 (emphasis in original). This idea fits with English legal history, in which judges could not exercise power sitting alone. Single judges could take verdicts, but judgment could only be entered by all judges of the court sitting together: see *Re Jarman; Ex parte Cook (No 1)* (1997) 188 CLR 595, 609–10.

14 Waldron (n 13) 25.

15 See Ronald Dworkin, *Law’s Empire* (Hart Publishing 1998) 167–75.

16 See sources cited in Nina Varsava, ‘How to Realize the Value of Stare Decisis: Options for Following Precedent’ (2018) 30 Yale LJ 62, 71 fn 17.

17 Greenberg (n 1) 1316–17.

18 Peter Westen, ‘The Empty Idea of Equality’ (1982) 95 Harv L Rev 537; Lyons (n 7); Larry Alexander, ‘Constrained by Precedent’ (1989) 63 S Cal L Rev 1, 10–13; Frederick Schauer, ‘Precedent’ in Marmor (ed), *Routledge Companion to Philosophy of Law* (n 1) 132; Neil Duxbury, *The Nature and Authority of Precedent* (CUP 2008) 170–5.

19 Grant Lamond, ‘Precedent and Analogy in Legal Reasoning’ in *Stanford Encyclopedia of Philosophy* (Edward N Zalta ed, 2016).

20 Duxbury (n 18) 172.

21 See Joel Feinberg, ‘Noncomparative Justice’ (1974) 83 Philosophical Review 297, 318–22.

22 Grant Lamond, ‘Precedent’ (2007) 2 Philosophy Compass 699, 707; Alexander (n 18) 10; Barbara Baum Levenbook, ‘Mark Greenberg on Legal Positivism’ in Torben Spaak and Patricia Mindus (eds), *The Cambridge Companion to Legal Positivism* (CUP 2021) 758; Adam Perry, ‘Consistency in Administrative Law’ in Endicott, Kristjánsson and Lewis (n 2) 492.

23 For the view that fairness always has some value, even if not decisive, see Neil MacCormick, ‘Formal Justice and the Form of Legal Arguments’ (1976) 73 Logique et Analyse 103, 109.

24 This is similar to the view that a world in which everyone is blind is better in some way—namely, in terms of equality—than a world in which only some people are blind. The value of this equality is only small, though: the first world is worse overall. See Larry Temkin, ‘Equality, Priority, and the Levelling Down Objection’ in Matthew Clayton and Andrew Williams (eds), *The Ideal of Equality* (Palgrave MacMillan 2002) 155.

25 I thank an anonymous referee for pressing the example in this paragraph.

26 Feinberg (n 21) 319.

27 ibid.

28 ibid 320.

29 David A Strauss, ‘Must Like Cases Be Treated Alike?’ (2002) University of Chicago Public Law & Legal Theory Working Paper No 24, 20. Strauss adds that if we can give these good reasons, then the decision is not unfair. In line with what I have said above, I think it better to say that the decision remains unfair, but that it is justified all things considered.

30 See Sebastian Lewis, ‘Precedent and the Rule of Law’ (2021) 41 OJLS 873, 881–2.

31 It may be more valuable in commercial disputes, for example, where the viability of investments depends on a stable playing field: Stephen R Perry, ‘Judicial Obligation, Precedent and the Common Law’ (1987) 7 OJLS 215, 242; see also Andrei Marmor, ‘Should Like Cases Be Treated Alike?’ (2005) 11 Legal Theory 27, 34.

32 What it means for an earlier decision to be ‘wrong’ may depend on one’s views about the nature of law, but the point does not matter here. I see legal matters as moral matters, so a decision is wrong if it incorrectly identifies or weighs the morally relevant factors. For a positivist view which instead distinguishes legal from moral incorrectness, see Sebastian Lewis, ‘Stare Decisis and Equitable Power’ [2023] Law and Philosophy.

33 Hershovitz, ‘Stare Decisis’ (n 3) 104; see also, Lamond, ‘Precedent’ (n 22) 702.

34 Grant Lamond, ‘Do Precedents Create Rules?’ (2005) 11 Legal Theory 1, 13.

35 The same view is given by Lewis, ‘Precedent and the Rule of Law’ (n 30) 886; Frederick Schauer, *Playing by the Rules* (Clarendon Press 1991) 142. There may be situations in which predictability and stability do not matter at all, but I suspect these are (very) rare. One example is discussed in Hillary Nye, ‘Predictability and Precedent’ in Endicott, Kristjánsson and Lewis (n 2).

36 Marmor (n 31) 28–9.

37 ibid 29.

38 ibid 29–30.

39 See *Wong v The Queen* (2001) 207 CLR 584, 611 [74]–[75].

40
*La Macchia v Minister for Primary Industries and Energy* (1992) 110 ALR 201, 204 (Burchett J).

41 ibid.

42
*Allergan Inc v Canada (Minister of Health)*, 2012 FCA 308, [47].

43 ibid [48]; *Hicks v Minister for Immigration & Multicultural & Indigenous Affairs* [2003] FCA 757, [76].

44
*Shaw v Yarranova Pty Ltd* [2006] VSC 45, [67].

45
*Practice Statement (Judicial Precedent)* [1966] 3 All ER 77.

46 See *Austin v Southwark London Borough Council* [2011] 1 AC 355, 370 [26].

47
*Young v Bristol Aeroplane Company Ltd* [1944] KB 718, 723, 725–6. Note that UK judges at first instance do not consider themselves strictly bound by decisions made in courts of co-ordinate jurisdiction: *Metropolitan Police District Receiver v Croydon Corporation* [1956] 2 All ER 785, 788.

48
*Singh v New Zealand Police* [2021] NZCA 91, [14].

49
*Collector of Customs v Lawrence Publishing Co Ltd* [1986] 1 NZLR 404, 414; see also *Dahya v Dahya* [1991] 2 NZLR 150, 159–60.

50 This paragraph draws on some ideas found in Aileen Kavanagh, ‘The Constitutional Separation of Powers’ in David Dyzenhaus and Malcolm Thorburn (eds), *Philosophical Foundations of Constitutional Law* (OUP 2016); Jeremy Waldron, ‘Separation of Powers in Thought and Practice?’ (2013) 54 BCL Rev 433.

51 I thank an anonymous referee for pressing this point.

52 In federal states, this includes disputes between governments about their relative powers. See eg *Supreme Court Act*, RSC 1985, c S-26, s 35.1 (in Canada); *Judiciary Act 1903* (Cth), s 75(iv) (in Australia); United States Constitution, art 3, §2.

53 For the fact that apex courts are more likely to spend their limited judicial resources on such questions, see eg *R v Gardiner* [1983] 2 SCR 368, 404, quoted with approval in *R v Shea* [2010] 2 SCR 17, 23 [9]; *Judiciary Act 1903* (Cth), s 35A(a)(ii).

54 Of course, not all superior courts are apex courts. Intermediate appellate courts may be less likely to deal with constitutional matters, but will likely spend their time on matters which have generated disagreement further below. This makes much of the discussion here relevant to those courts as well. Decisions of intermediate courts otherwise raise few questions for theories of precedent: they more often serve to correct errors in lower courts, rather than lay down new principles to be followed.

55 See Dworkin, *Law’s Empire* (n 15) 167–75. Dworkin puts the terms together (‘state or community’) at 167 and 168.

56 ibid 168.

57 ibid 172.

58 ibid.

59 ibid 171.

60 ibid 175.

61 Hershovitz, ‘Stare Decisis’ (n 3) 115.

62 ibid (emphasis added).

63 Hershovitz, ‘End of Jurisprudence’ (n 1) 1203 fn 91.

64 Is the clerk simply mistaken about what is required here? Perhaps she should simply apply the relevant provisions of the federal constitution and award the licence. But she also may face further facts which make it appropriate for her to deny the licence. For example, there may be previous decisions of the District Court denying the grant of such licences, and it may be appropriate for only the District Court itself to overrule those decisions.

65 Lewis thinks any account of precedent which focuses on moral reasons will face this objection: Lewis, ‘Nature’ (n 2) 47. I hope my reply to Lewis’ objection here is a convincing one.

66 Again, I thank an anonymous referee for pressing this Dworkinian line of thought.

67 Lewis, ‘Nature’ (n 2) 41.

68 JL Mackie, *Ethics: Inventing Right and Wrong* (Penguin 1990) 205.

69 Immanuel Kant, *The Moral Law: Groundwork of the Metaphysic of Morals* (HJ Paton tr, Routledge 2005) 74. For details of the critique, see Allen W Wood, ‘Kant’s Formulations of the Moral Law’ in Graham Bird (ed), *A Companion to Kant* (Blackwell 2006) 295ff.

70 Mark Greenberg, ‘The Standard Picture and Its Discontents’ in Leslie Green and Brian Leiter (eds), *Oxford Studies in Philosophy of Law*, vol I (OUP 2011) 74–5.

71 Hershovitz, ‘Stare Decisis’ (n 3) 104–6 discusses (and rejects) this argument.

